# *N*-(3,5-Dichloro­phen­yl)benzene­sulfonamide

**DOI:** 10.1107/S1600536808034351

**Published:** 2008-10-25

**Authors:** B. Thimme Gowda, Sabine Foro, K. S. Babitha, Hartmut Fuess

**Affiliations:** a Department of Chemistry, Mangalore University, Mangalagangotri 574 199, Mangalore, India; b Institute of Materials Science, Darmstadt University of Technology, Petersenstrasse 23, D-64287 Darmstadt, Germany

## Abstract

In the crystal structure of the title compound, C_12_H_9_Cl_2_NO_2_S, the aromatic rings are aligned at 57.0 (1)°. The mol­ecules form chains *via* inter­molecular N—H⋯O hydrogen bonds.

## Related literature

For the structural systematics of 4,4′-disubstituted aryl benzene­sulfonamides, see: Gelbrich *et al.* (2007 ▶). For mono- and di-substituted-aryl benzene­sulfonamides, see: Gowda *et al.* (2008*a* ▶,*b* ▶); Tkachev *et al.* (2006 ▶). For the spectroscopic analysis of the title compound, see: Shetty & Gowda (2005 ▶).
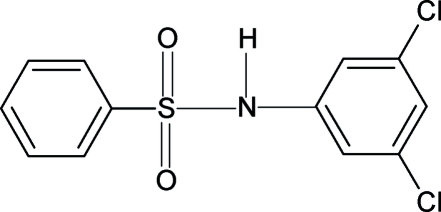


## Experimental

### 

#### Crystal data

C_12_H_9_Cl_2_NO_2_S
*M* *_r_* = 302.16
Monoclinic, 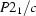

*a* = 8.299 (2) Å
*b* = 7.215 (1) Å
*c* = 21.954 (3) Å
β = 99.49 (1)°
*V* = 1296.6 (4) Å^3^
*Z* = 4
Cu *K*α radiation
μ = 5.96 mm^−1^
*T* = 299 (2) K
0.50 × 0.50 × 0.25 mm

#### Data collection

Enraf–Nonius CAD-4 diffractometer
Absorption correction: ψ scan (North *et al.*, 1968 ▶) *T*_min_ = 0.129, *T*_max_ = 0.229
2518 measured reflections
2311 independent reflections
2153 reflections with *I* > 2σ(*I*)
*R*_int_ = 0.050
3 standard reflections frequency: 120 min intensity decay: 1.0%

#### Refinement

*R*[*F* ^2^ > 2σ(*F* ^2^)] = 0.057
*wR*(*F* ^2^) = 0.156
*S* = 1.10
2311 reflections
167 parameters
1 restraint
H atoms treated by a mixture of independent and constrained refinement
Δρ_max_ = 0.59 e Å^−3^
Δρ_min_ = −0.38 e Å^−3^

### 

Data collection: *CAD-4-PC* (Enraf–Nonius, 1996 ▶); cell refinement: *CAD-4-PC*; data reduction: *REDU4* (Stoe & Cie, 1987 ▶); program(s) used to solve structure: *SHELXS97* (Sheldrick, 2008 ▶); program(s) used to refine structure: *SHELXL97* (Sheldrick, 2008 ▶); molecular graphics: *PLATON* (Spek, 2003 ▶); software used to prepare material for publication: *SHELXL97*.

## Supplementary Material

Crystal structure: contains datablocks I, global. DOI: 10.1107/S1600536808034351/ng2501sup1.cif

Structure factors: contains datablocks I. DOI: 10.1107/S1600536808034351/ng2501Isup2.hkl

Additional supplementary materials:  crystallographic information; 3D view; checkCIF report

## Figures and Tables

**Table 1:** Hydrogen-bond geometry (Å, °)

*D*—H⋯*A*	*D*—H	H⋯*A*	*D*⋯*A*	*D*—H⋯*A*
N1—H1*N*⋯O1^i^	0.856 (10)	2.059 (11)	2.915 (3)	178 (3)

Symmetry code: (i) 
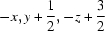
.

**Fig. 1.**
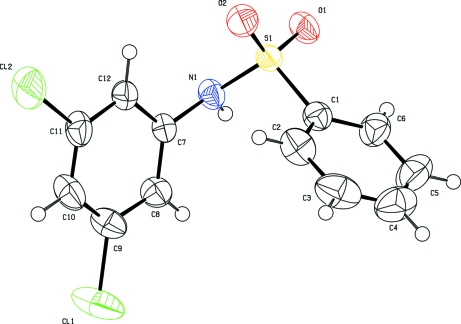
Molecular structure of the title compound, showing the atom labeling scheme. The displacement ellipsoids are drawn at the 50% probability level. The H atoms are represented as small spheres of arbitrary radii.

**Fig. 2.**
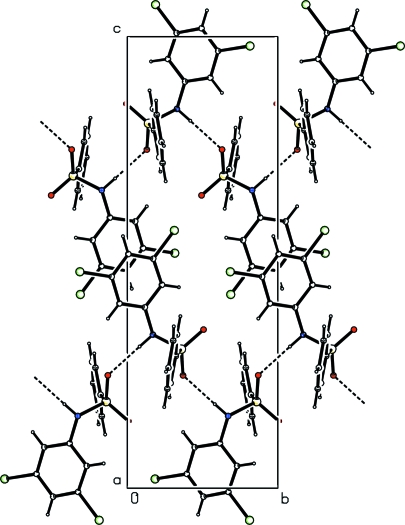
Molecular packing of the title compound with hydrogen bonding shown as dashed lines.

## supplementary crystallographic information

### Comment

As part of a study of the substituent effects on the crystal structures of *N*-(aryl)-benzenesulfonamides (Gowda *et al.*, 2008a,b), in the present work, the structure of *N*-(3,5-dichlorophenyl)- benzenesulfonamide (N35DCPBSA) has been determined. The conformations of the N—H and S═O bonds in N35DCPBSA are *trans* to each other (Fig.1), similar to that observed in *N*-(3-chlorophenyl)- benzenesulfonamide (N3CPBSA) (Gowda *et al.*, 2008b). The two benzene rings in N35DCPBSA form a dihedral angle of 57.0 (1)°, compared with the value of 65.4 (1)° in N3CPBSA (Gowda *et al.*, 2008b). The other bond parameters in N35DCPBSA are also similar to those observed in N3CPBSA and other *N*-(aryl)-benzenesulfonamides (Gelbrich *et al.*, 2007; Gowda *et al.*, 2008a,b; Tkachev *et al.*, 2006). The packing diagram of N35DCPBSA showing the N—H···O hydrogen bonds (Table 1) is shown in Fig. 2.

### Experimental

The solution of benzene (10 cc) in chloroform (40 cc) was treated dropwise with chlorosulfonic acid (25 cc) at 0 ° C. After the initial evolution of hydrogen chloride subsided, the reaction mixture was brought to room temperature and poured into crushed ice in a beaker. The chloroform layer was separated, washed with cold water and allowed to evaporate slowly. The residual benzenesulfonylchloride was treated with 3,5-dichloroaniline in the stoichiometric ratio and boiled for ten minutes. The reaction mixture was then cooled to room temperature and added to ice cold water (100 cc). The resultant solid *N*-(3,5-dichlorophenyl)-benzenesulfonamide was filtered under suction and washed thoroughly with cold water. It was then recrystallized to constant melting point from dilute ethanol. The purity of the compound was checked and characterized by recording its infrared and NMR spectra (Shetty & Gowda, 2005). The single crystals used in X-ray diffraction studies were grown in ethanolic solution by evaporating it at room temperature.

### Refinement

The H atom of the NH group was located in a diffrerence map and later restrained to the distance 0.86 (1) Å

The other H atoms were positioned with idealized geometry using a riding model with C—H = 0.93 Å. All H atoms were refined with isotropic displacement parameters (set to 1.2 times of the *U*_eq_ of the parent atom).

### Crystal data

**Table:**

C_12_H_9_Cl_2_NO_2_S	*F*(000) = 616
*M**_r_* = 302.16	*D*_x_ = 1.548 Mg m^−^^3^
Monoclinic, *P*2_1_/*c*	Cu *K*α radiation, λ = 1.54180 Å
Hall symbol: -P 2ybc	Cell parameters from 25 reflections
*a* = 8.299 (2) Å	θ = 5.4–19.4°
*b* = 7.215 (1) Å	µ = 5.96 mm^−^^1^
*c* = 21.954 (3) Å	*T* = 299 K
β = 99.49 (1)°	Prism, colourless
*V* = 1296.6 (4) Å^3^	0.50 × 0.50 × 0.25 mm
*Z* = 4	

### Data collection

**Table:**

Enraf–Nonius CAD-4 diffractometer	2153 reflections with *I* > 2σ(*I*)
Radiation source: fine-focus sealed tube	*R*_int_ = 0.050
graphite	θ_max_ = 67.0°, θ_min_ = 4.1°
ω/2θ scans	*h* = −9→1
Absorption correction: ψ scan (North *et al.*, 1968)	*k* = 0→8
*T*_min_ = 0.129, *T*_max_ = 0.229	*l* = −26→26
2518 measured reflections	3 standard reflections every 120 min
2311 independent reflections	intensity decay: 1.0%

### Refinement

**Table:**

Refinement on *F*^2^	Secondary atom site location: difference Fourier map
Least-squares matrix: full	Hydrogen site location: inferred from neighbouring sites
*R*[*F*^2^ > 2σ(*F*^2^)] = 0.057	H atoms treated by a mixture of independent and constrained refinement
*wR*(*F*^2^) = 0.156	*w* = 1/[σ^2^(*F*_o_^2^) + (0.0987*P*)^2^ + 0.7373*P*] where *P* = (*F*_o_^2^ + 2*F*_c_^2^)/3
*S* = 1.10	(Δ/σ)_max_ = 0.001
2311 reflections	Δρ_max_ = 0.59 e Å^−^^3^
167 parameters	Δρ_min_ = −0.38 e Å^−^^3^
1 restraint	Extinction correction: SHELXL97 (Sheldrick, 2008), Fc^*^=kFc[1+0.001xFc^2^λ^3^/sin(2θ)]^-1/4^
Primary atom site location: structure-invariant direct methods	Extinction coefficient: 0.0105 (11)

### Special details

**Table:**

Geometry. All e.s.d.'s (except the e.s.d. in the dihedral angle between two l.s. planes) are estimated using the full covariance matrix. The cell e.s.d.'s are taken into account individually in the estimation of e.s.d.'s in distances, angles and torsion angles; correlations between e.s.d.'s in cell parameters are only used when they are defined by crystal symmetry. An approximate (isotropic) treatment of cell e.s.d.'s is used for estimating e.s.d.'s involving l.s. planes.
Refinement. Refinement of *F*^2^ against ALL reflections. The weighted *R*-factor *wR* and goodness of fit *S* are based on *F*^2^, conventional *R*-factors *R* are based on *F*, with *F* set to zero for negative *F*^2^. The threshold expression of *F*^2^ > σ(*F*^2^) is used only for calculating *R*-factors(gt) *etc*. and is not relevant to the choice of reflections for refinement. *R*-factors based on *F*^2^ are statistically about twice as large as those based on *F*, and *R*- factors based on ALL data will be even larger.

### Fractional atomic coordinates and isotropic or equivalent isotropic displacement parameters (Å^2^)

**Table:**

	*x*	*y*	*z*	*U*_iso_*/*U*_eq_	
C1	0.3043 (3)	0.1779 (4)	0.79280 (13)	0.0445 (6)	
C2	0.4358 (4)	0.1697 (5)	0.84058 (17)	0.0604 (8)	
H2	0.4201	0.1476	0.8809	0.072*	
C3	0.5911 (4)	0.1953 (6)	0.8269 (2)	0.0783 (12)	
H3	0.6811	0.1914	0.8584	0.094*	
C4	0.6130 (4)	0.2267 (6)	0.7670 (2)	0.0773 (11)	
H4	0.7180	0.2427	0.7582	0.093*	
C5	0.4818 (5)	0.2344 (5)	0.7202 (2)	0.0746 (10)	
H5	0.4980	0.2547	0.6798	0.090*	
C6	0.3257 (4)	0.2121 (4)	0.73293 (15)	0.0562 (7)	
H6	0.2360	0.2200	0.7015	0.067*	
C7	0.1252 (3)	0.3838 (4)	0.90059 (11)	0.0400 (6)	
C8	0.2051 (4)	0.5522 (4)	0.90920 (13)	0.0484 (6)	
H8	0.2132	0.6288	0.8758	0.058*	
C9	0.2725 (4)	0.6041 (4)	0.96827 (15)	0.0558 (7)	
C10	0.2645 (4)	0.4935 (4)	1.01861 (14)	0.0590 (8)	
H10	0.3122	0.5293	1.0582	0.071*	
C11	0.1830 (4)	0.3278 (4)	1.00819 (13)	0.0534 (7)	
C12	0.1118 (3)	0.2699 (4)	0.95062 (12)	0.0480 (6)	
H12	0.0561	0.1577	0.9451	0.058*	
N1	0.0494 (3)	0.3297 (3)	0.84009 (10)	0.0441 (5)	
H1N	0.038 (4)	0.418 (3)	0.8138 (12)	0.053*	
O1	−0.0021 (3)	0.1298 (3)	0.74998 (9)	0.0527 (5)	
O2	0.1112 (3)	−0.0066 (3)	0.85135 (10)	0.0574 (6)	
Cl1	0.37237 (16)	0.81541 (13)	0.97944 (5)	0.0927 (4)	
Cl2	0.16462 (15)	0.18752 (15)	1.07089 (4)	0.0834 (4)	
S1	0.10584 (7)	0.14095 (9)	0.80800 (3)	0.0412 (3)	

### Atomic displacement parameters (Å^2^)

**Table:**

	*U*^11^	*U*^22^	*U*^33^	*U*^12^	*U*^13^	*U*^23^
C1	0.0475 (14)	0.0400 (13)	0.0467 (15)	0.0019 (11)	0.0101 (11)	0.0007 (11)
C2	0.0571 (17)	0.0615 (19)	0.0589 (19)	0.0079 (14)	−0.0009 (14)	−0.0049 (15)
C3	0.0517 (18)	0.065 (2)	0.111 (3)	0.0057 (15)	−0.0071 (19)	−0.014 (2)
C4	0.0560 (19)	0.063 (2)	0.117 (4)	0.0031 (16)	0.029 (2)	0.001 (2)
C5	0.079 (2)	0.064 (2)	0.091 (3)	−0.0014 (18)	0.044 (2)	0.0114 (19)
C6	0.0619 (17)	0.0556 (17)	0.0535 (17)	0.0004 (14)	0.0161 (13)	0.0070 (13)
C7	0.0447 (13)	0.0413 (13)	0.0349 (12)	0.0025 (10)	0.0088 (10)	−0.0039 (10)
C8	0.0581 (15)	0.0404 (14)	0.0465 (14)	0.0000 (12)	0.0079 (12)	0.0033 (12)
C9	0.0631 (17)	0.0424 (15)	0.0585 (17)	−0.0015 (13)	0.0001 (13)	−0.0043 (13)
C10	0.078 (2)	0.0531 (17)	0.0426 (15)	0.0025 (15)	0.0004 (14)	−0.0087 (13)
C11	0.0701 (18)	0.0540 (16)	0.0379 (14)	0.0049 (14)	0.0141 (13)	0.0020 (12)
C12	0.0593 (16)	0.0466 (15)	0.0405 (14)	−0.0057 (12)	0.0157 (12)	−0.0028 (11)
N1	0.0547 (13)	0.0423 (12)	0.0350 (12)	0.0020 (10)	0.0064 (9)	−0.0004 (9)
O1	0.0548 (11)	0.0586 (12)	0.0427 (11)	−0.0059 (9)	0.0021 (8)	−0.0107 (9)
O2	0.0838 (15)	0.0415 (11)	0.0499 (11)	−0.0053 (10)	0.0199 (10)	0.0058 (8)
Cl1	0.1209 (9)	0.0518 (5)	0.0915 (8)	−0.0250 (5)	−0.0236 (6)	−0.0022 (4)
Cl2	0.1333 (9)	0.0798 (7)	0.0393 (5)	−0.0122 (6)	0.0204 (5)	0.0085 (4)
S1	0.0495 (4)	0.0384 (4)	0.0359 (4)	−0.0042 (2)	0.0076 (3)	−0.0018 (2)

### Geometric parameters (Å, °)

**Table:**

C1—C6	1.377 (4)	C7—N1	1.427 (3)
C1—C2	1.385 (4)	C8—C9	1.377 (4)
C1—S1	1.754 (3)	C8—H8	0.9300
C2—C3	1.383 (5)	C9—C10	1.374 (5)
C2—H2	0.9300	C9—Cl1	1.734 (3)
C3—C4	1.376 (6)	C10—C11	1.374 (5)
C3—H3	0.9300	C10—H10	0.9300
C4—C5	1.370 (6)	C11—C12	1.369 (4)
C4—H4	0.9300	C11—Cl2	1.736 (3)
C5—C6	1.379 (5)	C12—H12	0.9300
C5—H5	0.9300	N1—S1	1.637 (2)
C6—H6	0.9300	N1—H1N	0.856 (10)
C7—C8	1.382 (4)	O1—S1	1.433 (2)
C7—C12	1.391 (4)	O2—S1	1.424 (2)
			
C6—C1—C2	121.4 (3)	C7—C8—H8	120.7
C6—C1—S1	118.8 (2)	C10—C9—C8	122.3 (3)
C2—C1—S1	119.8 (2)	C10—C9—Cl1	118.9 (2)
C3—C2—C1	118.5 (4)	C8—C9—Cl1	118.9 (2)
C3—C2—H2	120.8	C11—C10—C9	117.3 (3)
C1—C2—H2	120.8	C11—C10—H10	121.3
C4—C3—C2	120.3 (4)	C9—C10—H10	121.3
C4—C3—H3	119.9	C12—C11—C10	123.0 (3)
C2—C3—H3	119.9	C12—C11—Cl2	118.2 (2)
C5—C4—C3	120.6 (3)	C10—C11—Cl2	118.7 (2)
C5—C4—H4	119.7	C11—C12—C7	118.0 (3)
C3—C4—H4	119.7	C11—C12—H12	121.0
C4—C5—C6	120.0 (4)	C7—C12—H12	121.0
C4—C5—H5	120.0	C7—N1—S1	120.98 (18)
C6—C5—H5	120.0	C7—N1—H1N	114 (2)
C1—C6—C5	119.2 (3)	S1—N1—H1N	110 (2)
C1—C6—H6	120.4	O2—S1—O1	119.87 (14)
C5—C6—H6	120.4	O2—S1—N1	108.29 (12)
C8—C7—C12	120.7 (2)	O1—S1—N1	104.34 (12)
C8—C7—N1	119.7 (2)	O2—S1—C1	108.30 (14)
C12—C7—N1	119.5 (2)	O1—S1—C1	107.97 (13)
C9—C8—C7	118.6 (3)	N1—S1—C1	107.45 (13)
C9—C8—H8	120.7		
			
C6—C1—C2—C3	−0.5 (5)	C10—C11—C12—C7	1.0 (5)
S1—C1—C2—C3	178.6 (3)	Cl2—C11—C12—C7	179.2 (2)
C1—C2—C3—C4	−0.5 (5)	C8—C7—C12—C11	−1.4 (4)
C2—C3—C4—C5	0.5 (6)	N1—C7—C12—C11	−178.6 (3)
C3—C4—C5—C6	0.5 (6)	C8—C7—N1—S1	119.8 (2)
C2—C1—C6—C5	1.5 (5)	C12—C7—N1—S1	−62.9 (3)
S1—C1—C6—C5	−177.6 (3)	C7—N1—S1—O2	48.5 (2)
C4—C5—C6—C1	−1.5 (5)	C7—N1—S1—O1	177.3 (2)
C12—C7—C8—C9	0.6 (4)	C7—N1—S1—C1	−68.3 (2)
N1—C7—C8—C9	177.8 (3)	C6—C1—S1—O2	138.8 (2)
C7—C8—C9—C10	0.7 (5)	C2—C1—S1—O2	−40.3 (3)
C7—C8—C9—Cl1	−179.8 (2)	C6—C1—S1—O1	7.6 (3)
C8—C9—C10—C11	−1.2 (5)	C2—C1—S1—O1	−171.5 (2)
Cl1—C9—C10—C11	179.4 (3)	C6—C1—S1—N1	−104.5 (2)
C9—C10—C11—C12	0.3 (5)	C2—C1—S1—N1	76.5 (3)
C9—C10—C11—Cl2	−178.0 (3)		

### Hydrogen-bond geometry (Å, °)

**Table:**

*D*—H···*A*	*D*—H	H···*A*	*D*···*A*	*D*—H···*A*
N1—H1N···O1^i^	0.86 (1)	2.06 (1)	2.915 (3)	178 (3)

Symmetry codes: (i) −*x*, *y*+1/2, −*z*+3/2.
